# Statistical Reports on the Sickness, Mortality, and Invaliding among the Troops in Western Africa, St. Helena, the Cape of Good Hope, and the Mauritius; Prepared from the Records of the Army Medical Department and War-Office Returns. Presented to Both Houses of Parliament by Command of Her Majesty

**Published:** 1841-04

**Authors:** 


### Art. IV.

1. *Statistical Reports on the Sickness, Mortality, and Invaliding among the Troops in Western Africa, St. Helena, the Cape of Good Hope, and the Mauritius; prepared from the Records of the Army Medical Department and War-office Returns. Presented to both Houses of Parliament by Command of her Majesty.—London,* 1840. Folio.

2. *Traité des Maladies des Européens dans les Pays chauds, et spécialement au Sénégal; ou Essai Statistique Médical et Hygiènique sur le Sol, le Climat, et les Maladies de cette partie de l'Afrique.* Par J. P. F. Thevenot, Chirurgien de première Classe de la Marine, &c. *Publié par Ordre de M. le Ministre de la Marine et des Colonies.—Paris,* 1840. 8vo, pp. 399.

*Treatise on the Diseases of Europeans in Warm Countries, and especially in Senegal; or a Statistical, Medical, and Hygienic Essay on the Soil, the Climate, and the Diseases of that part of Africa.* By J. P. F. Thevenot, Surgeon of the First Class of the Navy, &c. *Published by Order of the Minister of the Navy and the Colonies.—Paris,* 1840.

3. *Practical Medico-Historical Account of the Western Coast of Africa; embracing a Topographical Description of its Shores, Rivers, and Settlements, with the Causes, Symptoms, and Treatment of the Fevers of Western Africa. A similar Account respecting the other Diseases which prevail there.* By James Boyle, M.C.S.L., Colonial Surgeon to Sierra Leone, Surgeon in the Royal Navy.—*London,* 1831. 8vo, pp. 423.

We have pleasure in presenting our readers with another valuable Report from the pen of Major Tulloch. The volumes which we have associated with it relate to the climate and diseases of the district forming the subject of the first part of this Report, the western coast of Africa; and as they are both of them the works of medical men, they constitute, as might be expected, valuable supplements to the more strictly statistical work of the gallant Major.

The British settlements in Western Africa are Sierra Leone, Gambia, the Isles de Loss, Cape Coast Castle, and Accra. They are scattered over a line of coast, which, from St. Mary's on the Gambia to Accra, is nearly 1600 miles in extent, and, consequently, presents considerable diversity of climate, soil, surface, and geological structure, but everywhere exhibits the same remarkable hostility to the European constitution. The coast is generally low; its elevation from Senegal to Sierra Leone, a distance of 100 miles, being only a few feet above the ocean; its rivers are sluggish and flooded during rains, when the mud they deposit and the moisture they supply give rise to an interminable wilderness of forest and brushwood, among which lies rotting the decayed vegetation of many centuries.

In the Sierra Leone command are comprised the stations of Sierra Leone, the Isles de Loss, and the Gambia. The peninsula of Sierra Leone occupies an intermediate position in our settlements along this coast, being about 500 miles to the south of the Gambia, and 1100 to the north of Accra, and comprehends a tract of land extending about eighteen miles from north to south, and twelve from east to west, consisting principally of a range of conical mountains from 2000 to 3000 feet in height, surrounded by a belt of level ground from one to five miles in breadth.

From the noxious agencies likely to be generated in such a tract as has been described as extending from Senegal to this colony, it is sheltered by the mountain ranges which form the boundary in that direction, while on the south it is washed by the Atlantic, and on the north by an estuary terminating in the Sierra Leone river; so that it seems protected by nature from all extraneous sources of disease, except such as may originate on the opposite side of the river, called the Bulam shore. There is nothing in the nature of the soil of the colony, as described by Mr. Boyle, which, according to received opinion, is calculated to render it prejudicial to health. He says, "in the direction of the mountains it is generally thin, arid, and pebbly, with occasional large granite rocks projecting; and in the lower parts it is ordinarily either light unproductive sand, or a superficial and equally barren stratum of dark loam." Free Town, the capital of the colony, is so built as to comprise nothing in its structure noxious to health. The streets are fifty or sixty feet in width; the principal houses have rooms of good dimensions, lofty, and of a large area; the kitchens and other domestic offices are detached from the residence, and their whole arrangement seems well fitted for ventilation and comfort. The inferior dwellings, called frame-houses, are mostly detached, with yards or small gardens, and so placed as to afford free ventilation. The Bulam shore opposite to it has a soil of a ferruginous clayey loam, apt to form marshes during the rainy season, owing to the surface being almost level; but this shore is distant about seven miles. Mangrove bushes, which formerly covered the banks of the river with a dense barrier, and were supposed to be a fertile source of disease, have been cleared to a considerable extent, without, however, effecting any manifest improvement in salubrity. The barracks for the troops are stated to be good, ample, and commodious; they are situated on the top of a conical hill, 400 feet high, and nearly in the centre of the elevated range which surrounds Free Town. It would appear then, that, independent of a hot and an excessively wet climate, the acknowledged sources of disease do not abound immediately at Sierra Leone. Neither would they appear to do so at the Isles de Loss, for Crawford's island, where the troops for the defence of these isles are stationed, is a granite rock, elevated 250 feet above the level of the sea, and is said to be

"…. entirely exempt from that exuberance of vegetation which prevails on the main land, and in no part of it, nor in any of the other islands, are either pools or marshes to be found; so that if the insalubrity of the command arose from the combined influence of these agencies, it ought not to have extended to this station, unless we can suppose the miasma capable of being conveyed from the main land with undiminished virulence across several miles of ocean.

"The principal settlement on the Gambia is at the island of St. Mary's, about 500 miles N. W. of Sierra Leone. It lies in lat. 13° 25″ N., long. 16° 38″ W., is five miles in length and one in breadth, and consists merely of a sand-bank formed by the confluence of the tides at the mouth of the river. It is consequently, in many places, under high-water mark, which renders it during the rainy season one complete marsh. The soil is too light to be well fitted for agriculture, but where intersected by creeks, and aided by the alluvial deposits brought down by the river, gives birth to dense masses of mangroves, under-wood, and every species of rank vegetation, which, particularly during the hot season, create most offensive effluvia throughout the whole island. The country along the banks of the river, to the distance of fifty or sixty miles from its mouth, is so low as to be nearly on a level with the water, is covered with im-penetrable mangrove bushes, reeds, and brushwood, and at ebb-tide innu-merable shoals of mud and masses of decayed vegetation are left exposed to the action of a tropical sun." (Tulloch's Western Africa, p. 4.)

The principal characteristic of the climate of the western coast of Africa, we are informed, is its extreme humidity. As an illustration of this, Major Tulloch furnishes us with a table of rain which fell at Sierra Leone during the months of June, July, and August, 1828; it amounted to the enormous quantity of 313·4 inches: but this was an unusually wet season; and we are presented with another table, according to which 144·54 inches fell during six months (from July to December) of 1819. The rain does not fall with any degree of uniformity throughout the year; for instance, whilst July has above forty-five and August above forty-six inches of rain, there are but five inches in November and six in January. The wet season, we are informed, extends at Sierra Leone and the Isles de Loss from May to November, and at the Gambia from June to September or October, and is always ushered in and car-ried off by tornados.

In note A. of the Appendix to this Report, Major Tulloch adduces evidence to show the moisture of the atmosphere of Western Africa from what may be called natural hygrometers, which appear to be of a very conclusive kind. He remarks very justly, that

"The moisture of the atmosphere depends in part on the quantity of rain which falls, and partly upon circumstances which retard evaporation. In the torrid zone the quantity of vapour contained in the air is much nearer the point of saturation than in the temperate zone; in consequence of which the evapo-ration is much less than might be supposed from the high temperature. Various instruments," he adds, "have been invented to measure the degree of moisture in the atmosphere, but, in practice, these instruments seem to indicate the amount of evaporation rather than the absolute moisture. In the absence of a more accurate standard, some idea may be formed of the extent to which the latter exists in tropical climates by its influence on inorganic bodies. For example: 1. The rapid oxygenation of iron, by which tinned vessels are soon covered with rust, and iron fastenings, unless of considerable strength, are soon reduced to a state of powder; 2, the effect on common salt, which, unless carefully excluded from the atmosphere, speedily dissolves; 3, the effect on glue and paste, which soon lose their tenacious qualities—for articles of furniture in which the former has been used fall to pieces, and paper, though well sized, becomes deteriorated and unfit for use; 4, the destruction of the texture and colour of cloth—for some kinds, particularly woollens, are soon covered with spots, and, unless frequently dried, become rotten; 5, the mouldiness of leather, for, during the wet season, in some tropical climates, boots and shoes become covered with mould in one night; 6, the rapid destruction of soft wood, which, if left underground, will rot and fall to powder within a year; 7, the rapid putrefaction of animal and rapid fermentation of vegetable substances. All these indications of extreme moisture are particularly observable in the climate of Western Africa during the rainy season." (Tulloch, Note A of Appendix to Report on Western Africa.)

The temperature, as stated in the table for 1820, published in the report, is chiefly remarkable, both at Sierra Leone and Gambia, for its great uniformity. In the former colony we find the annual range of the thermometer to be but 12°, the extremes being 87° and 75°; whilst at Gambia it is shown to be but 14°, the extremes being 89° and 75°. A remark, however, of Major Tulloch's leads us to conclude that there must have been a peculiarity in 1820 as to temperature; for he says that, "with the exception of Sierra Leone, where the diurnal range of the thermometer rarely exceeds 10°, sudden transitions from heat to cold form the general characteristic of the whole west coast of Africa, particularly at the Gambia, where the thermometer has sometimes fallen as low as 62° in the morning during October, November, and December, and then risen to 80° in the course of a few hours."

The garrison of Sierra Leone has consisted, since 1829, chiefly of black troops, with a slight intermixture of white non-commissioned officers, the fatality of the climate to the European constitution having been by that time so clearly demonstrated as to induce the government to alter their mode of garrisoning the colony. For, though the mortality at Sierra Leone was less enormous than at the Gambia, which, as Major Tulloch expresses it, "proved the grave of almost every Englishman sent thither," it was yet too considerable to justify any government in retaining the colony at such a price. The admissions at Sierra Leone were 2978, and and the deaths 483 per thousand of the strength annually; in other words, every soldier was thrice under treatment, and nearly half of the force perished every year. Some corrections of this estimate required by the circumstance of many of the deaths in 1825 and 1826 having taken place at the Gambia reduce the mortality to 350 per thousand, or somewhat more than a third of the mean strength.

This place, the Gambia, is, as might be expected from the noxious influences in operation there, the most pernicious to European life of our colonies on this coast. The statement on this hand in the report is as brief, but, at the same time, as forcible as possible. A detachment of 108 men arrived in the colony in the latter end of May, 1825, just as the rains commenced. Between that date and the 21st of September of the same year, there died of remittent fever seventy-four, of other diseases thirteen, and there remained alive at the end of four months twenty-one. Another detachment, consisting of ninety-one, at this period landed towards the end of September, room being provided for them in the barracks by the death of four-fifths of their comrades. Of the force thus raised to 112, there died between that time and the 21st of December, of fever sixty-one, and of other diseases twelve, forming a total of seventy-three, and there remained alive thirty-nine. Another body of 200 Europeans was sent out to recruit the force thus reduced; it arrived, as before, at the commencement of the rainy season. The deaths reported from the 21st of June to 21st of September, 1826, were, from fever eighty-five, and from other causes thirteen, forming a total of ninety-eight. Eighteen more of the force, reduced by the previous mortality to 108, perished between the 21st of September and the 21st of December. Thirty-three of the survivors, who were suffering under chronic affections of the liver, spleen, and other viscera, were removed to head-quarters, and in June of the following year all the white troops were withdrawn from the station.

The following is a summary of the mortality at the Gambia. Between the end of May, 1825, and December, 1826, a period of only nineteen months, 279 perished out of a force, of which the number on shore seldom exceeded 120, and was sometimes even as low as 40. We are informed that, during the whole of this dreadful mortality, a detachment of from forty to fifty black soldiers of the second West India regiment lost only one man, and had seldom any in hospital.

We have already mentioned the circumstances in the geological formation and nature of the soil of this station, which gave it a character for salubrity which, unfortunately, was by no means justified by experience. Of a detachment of 103 men which landed there in the early part of 1825, in eighteen months sixty-two were dead and twenty-one invalided to England, consequently there remained of the original force but twenty; and such of these as survived were withdrawn at the close of the year, scarcely any of them being fit for duty. Major Tulloch makes the following estimate of the relative mortality at the respective stations during the years 1825 and 1826. At the Gambia there died 500 per thousand of the force employed, at Sierra Leone 650, and at the Isle de Loss 600; so that this quarter, though possessing some advantage over the other stations, still appears to labour under the ban apparently directed against the whole territory set apart for the descendants of Ham, that of being uninhabitable by the "genus Iapeti."

Fever is, as would be supposed, the cause of this frightful mortality, and remittent fever is at once its most prevalent and fatal form. A few extracts from the tables will show this. At Sierra Leone, the admissions from fever during eighteen years were 2600, and the deaths from the same cause 756; whilst the total admissions from all diseases (fevers included) were 5489, and the total deaths but 890; or, to state the ratio of deaths to the mean strength, whilst this is 410·2 per thousand from fevers only, it is but 483 per thousand from all diseases, fevers included. Then again, whilst of these 2,600 cases of fever 948 are intermittents, of which eleven are fatal, and sixty-one are common continued, of which six are fatal; 1601 are cases of remittent fevers, from which a mortality of 739 results; or, to state the fatality proportionally, intermittents kill one in eighty-six of those attacked, common continued fever one in eight and a half, and remittents one in two.

The enormous mortality from remittents of course produces a doubt as to the accuracy of the name given to a disease so fatal. Major Tulloch remarks that, except in two points which he mentions, there is little difference in the character of the disease from that which generally marks the course of yellow fever of the worst type in other colonies. These two points are, that ulcers, which are very common among soldiers who indulge in intemperance, seemed to act as a safeguard against remittent fever so long as they continued open and discharged freely, but whenever they ceased to do so that it assailed the patient, and generally proved fatal; and that, in 1837, it was observed that, in many of the worst cases, an exudation of blood took place from the tongue, gums, nose, and anus, and that, whenever leeches were applied, the tendency to hemorrhage was so great as to render it almost impossible to stop the effusion. With regard to the first point, we would remark that this prophylactic power of ulcers over tropical fevers has been noticed in other localities—the West Indies, for instance; and we cannot regard it as at all decisive of the character or correct designation of the fever presented. As respects the second point, we would observe that hemorrhage is one of the characteristics of yellow fever, as is shown in our review of Louis's account of the Gibraltar Epidemic; and, although it seems to have been more general and severe in the African disease, it should be observed that the African, however it is to be designated, seems to have been altogether a more malignant form of fever than that at Gibraltar. Major Tulloch remarks that the absence of all mention of black vomit in the reports between 1824 and 1829, when the disease raged in its most aggravated form along the whole coast for several months each year, may induce a doubt whether the disease was yellow fever or the endemical remittent of the country. In the first place, great accuracy in reports of symptoms could scarcely be expected under the circumstances in which medical officers would certainly be placed on the western coast of Africa; the surprise is, that there were any reports at all. Again, it was well ascertained during the Gibraltar epidemic of 1828 that yellow fever often exists, and is sometimes fatal, without black vomit.

Mr. Boyle, in his account of the fever at Freetown in 1829, leaves no doubt of *his* opinion of its nature, for he calls it the epidemic, Bulam, or *yellow* fever; and in his really interesting description of the progress of this fever through the town, black vomit is frequently mentioned among the symptoms, and yellowness of the skin is very generally noticed. We regret, but we are not surprised, that none of the writers favour us with autopsies, for we should have been glad to observe how far M. Louis's pathognomic distinction, the anæmious condition of the liver, was present or otherwise.

M. Thévenot seems to have no doubt regarding the nature of the fever we are considering; for he says,

"Yellow fever, which we may justly suppose to be endemic on the neighbouring shore, nevertheless does not pass certain bounds. *Almost permanent at Sierra Leone*, it is rare near the Senegal; and its having twice appeared on the arid sands of Goree cannot be ascribed to the ordinary circumstances of the soil and climate. The marshy countries which border on the Great Desert are the cradle of intermittent and remittent fevers, whilst yellow fever has its origin in the uncultivated marshes near the equator. Thus, the sands of Sahara, the action of the east wind, and the long continuance of dry weather, tend incessantly to diminish the action of the miasma exhaled from the soil, whilst longcontinued humidity favours it in the marshes lower down the coast." (Thévenot, p. 244.)

Major Tulloch's information tends considerably to confirm these views of M. Thévenot on the connexion between humidity and the prevalence of malignant fever in *our* African possessions; though with that fidelity and freedom from theoretic bias which characterize him as a reporter, he states several exceptions:

"The disease," he says, "has, in most years, appeared and raged with the greatest violence during the height of the rainy season, when vegetation was most vigorous and healthy, and the low grounds, being completely flooded, were in the state supposed least favorable to the extinction of miasma. It has also been observed to diminish both in prevalence and severity as the rains moderated, and the marshes began to dry; but to this rule we have to record several exceptions. For instance, in 1823, 1829, 1837, and 1838, the disease appeared in its most malignant form in the months of February and March, during what had generally been termed the dry or healthy season; and, on each of these occasions, its violence declined as the rainy season advanced, and the earth became completely saturated with moisture, being directly the reverse of what has been observed in other years.

"In most instances, too, the prevalence and fatal character of the disease have been commensurate with the quantity of rain; but here, again, the exceptions are so numerous as to strike completely at the root of all theories tending to establish that the former is a necessary result of the latter, for in 1812, 1823, and 1829, less rain fell than usual, yet fever was extremely prevalent; and though from 1830 to 1836 the colony was almost free from fever, yet, with the exception of 1832, the quantity of rain in each year was about the usual average." (Tulloch's Report, p. 9.)

This statement must prove exceedingly unsatisfactory to those to whom a state of doubt and uncertainty is insupportable. Nature, however, is slow to disclose the sources of disease; and researches, or rather conjectures, in other directions have been equally unsatisfactory.

"Attempts have also been made to connect the appearance of this disease with the circumstance of the rains commencing earlier or later, or being heavier or lighter at the commencement than usual, but without any satisfactory result, the exceptions being always too numerous to admit of any positive conclusions. The range of temperature, the fluctuations in the barometer, the direction and prevalence of the winds, have also been carefully observed, but do not seem in any way connected with it. The latest as well as the earliest observations all tend to show that the circumstances which call it into operation at one season, or in one year, more than another, have hitherto received no satisfactory explanation. In 1823, when the fever broke out during what was deemed the healthy season, and when none of the usual theories would account for its appearance, it was supposed by some of the medical officers to have originated in noxious miasma, wafted from the Bulam shore; but, so far as could be learnt, there was nothing in the character of that shore more likely to have produced the disease in 1823 than in other years which were healthy; and its subsequent appearance in 1837 and 1838 at the same season, when there was nothing to induce such a supposition as to its origin, certainly appears to indicate that there could be but little foundation for this theory." (Tulloch's Report, pp. 8-9.)

We believe that the sum of our information on this subject amounts to this, that *where* diseases, ordinarily considered to be the product of malaria exist sporadically, *there* are they liable at intervals to assume an epidemic character, without the causes which invest them with this disposition to diffusion being, in the present state of our knowledge, discernible. To say that this arises from these diseases then assuming a contagious character, is only shifting the difficulty; for what invests them with this character, of which we do not deny the existence? If we say that diseases which become occasionally epidemic move in cycles, this is merely another mode of expressing the same fact. When yellow fever which is observed to exist sporadically at Gibraltar, and other places along the Spanish coast, appears there in the form of a destructive epidemic, meteorological journals and geological formations are ransacked in vain for an explanation; whilst another class of disputants speculate in a way equally unsatisfactory about importation and unclean bills of health. In spite of these efforts, however, the pestilence still remains "the pestilence which walketh in darkness;" but if its path is ever to be illuminated, we will venture to say that it is rather by the former than the latter mode of investigation. What Dr. Rush said of America and its visitations of yellow fever is true of every country subject to these occasional pestilential inflictions, "She (America) must consent to consider it as engendered in her own bosom, or cease to rank among civilized nations."

Before considering the degree of prevalence and fatality of fever in the negro race in the climate just passed under review, it will be instructive to give an abstract of the general facts furnished in the reports, illustrative of their general health and longevity in a climate which might be expected to be congenial to them. The Royal African corps is recruited from the slaves captured by our cruizers and liberated at Sierra Leone. From 1819 to 1836 inclusive, their average annual strength was 421, the admissions into hospital 342, and the deaths per medical returns 12 $\frac{2}{3}$. The admissions stated in the ratio of 1000 of mean strength were 812 annually, and the deaths 30·1. If to this last ratio be added sudden and accidental deaths not stated in the medical returns, it will be increased in all probability to thirty-two per thousand. Major Tulloch compares this with that of the dragoon guards and dragoons in the United Kingdom, of Maltese fencible corps, of the Hottentot Cape corps, and of the Madras and Bengal native troops, and remarks that, at the lowest computation, the mortality of the black troops serving in the colony has been twice and, in some instances, more than thrice as high as among other troops serving in their native country; though, from the recent formation of the African corps, most of them are considerably under the average age of soldiers. The ratio is found to be exactly the same as among the black troops employed in Jamaica and Honduras, and, making some allowance for the difference in age of the men of the respective forces, corresponds very nearly with that observed among the similar force in the Bahamas and Windward and Leeward command.

Making every allowance for deterioration of constitution in the ill-ventilated holds of slave-ships, the mortality among these troops shows that the climate of Sierra Leone is by no means favorable even to the negro race, and this view is confirmed by the abstract of the mortality among the negro colonists not subjected to the restraints and discipline of a military life. Of liberated slaves landed between the formation of the colony and 1826 there arrived 17,883, of whom there were alive, including children, in 1826, 10,716, being a reduction of 7167; of Africans from Nova Scotia, landed in 1792, arrived 1131 alive, including children, in 1826, 578, decrease 553; Maroons from Jamaica, landed in 1800, arrived 550 alive, including children, in 1826, 636, increase 86; or, in about thirty years, a population of 19,564 suffered a diminution of 7634. This diminution may have arisen in part from emigration, and there is reason to believe that some of the liberated Africans have been again kidnapped and reduced to slavery; but, after every allowance for these possible contingencies, the reporter considers the climate nearly as unfavorable to the civil as the military portion of the negro population.

We regard this evidence of the insalubrity of this portion of the western coast of Africa to the negro race as conclusive; but we find by the returns that very little of the mortality which has effected this diminution of population has arisen from fevers. The annual ratio of admissions from these diseases has been but 54, and the deaths but 2·4 per 1000 of mean strength; or, as is stated in the report, the attacks have been fewer, and the deaths have not materially exceeded the proportion among an equal number of white troops in the United Kingdom, or other temperate climates. Though fevers are much more frequent and fatal, it is added, among the whites than in the West Indies, the reverse is the case with the blacks; for of black troops there died annually from fevers per 1000, in the Windward and Leeward Command, 4·6; in Jamaica, 8·2; Honduras, 4·4; Bahamas, 5·6; and in Sierra Leone, 2·4; so that it seems established that the susceptibility of the negro to febrile agency increases when removed from his native land.

Continuing to trace the mortality on this coast, and the influence of fever in contributing to it, we proceed to the second portion of the report or that relating to Cape Coast Command. Cape Coast Castle is 1000 miles to the E. and S. of Sierra Leone; it is built on a rock fifty feet high jutting into the sea, which is described as having a salt pond about a mile distant, but no swamps or marshes in the vicinity, nor any river nearer than five or six miles. The climate, as to humidity resembles that of Sierra Leone and Gambia. In this Command, two thirds of the white troops died annually, and the deaths from fevers amounted to 382·6 per 1000 of the mean strength annually.

A report of sickness and mortality among the officers serving in Western Africa, though from circumstances less full and accurate than those relating to the privates, tends to show that from fevers this class has suffered nearly in the same proportion as the soldiers, or, where the advantage has appeared to be on their side, this has arisen from the opportunity enjoyed by most of the officers of returning home, when their constitutions had become impaired by attacks of this disease. With regard to diseases of the bowels, a subject to which we shall subsequently advert, officers, as in the West Indies, enjoyed a comparative exemption; thus, as Major Tulloch remarks, inducing the inference that they must have arisen from some other cause than climatorial influence.

In the Fourth Section Major Tulloch presents his deductions from the Reports on Western Africa, and, examining the different causes to which this fever, whether correctly designated as remittent or yellow, has been ascribed by different investigators, regards successively excessive moisture, high temperature, variations in atmospheric pressure, and the prevalence of particular winds as unconnected with its origin. With regard to the effluvia of marshes he says:

"The hypothesis that this fever originates from the miasma of marshes in the immediate vicinity of the station, as elsewhere it has been supposed to do, is directly opposed to the fact of the Isles de Loss, Accra, and the peninsula of Sierra Leone itself, being so subject to it, though all are in a certain degree, remote from the operation of any such agency. If it be referred to similar exhalations wafted to the distance of several miles, how is its prevalence to be accounted for at Fernando Po, a mountainous region and bordering on a main land still more so, and where, so far as can be ascertained, no such agency is in operation? Instances of the disease having raged with the same violence on the rocky Isles de Loss and the sandy wastes of Senegal, as in those parts of the coast where vegetation is most dense, preclude the likelihood of it originating in a superabundance of that agency. In every description of situation along the coast has this scourge of Europeans been found to prevail. The low swampy Gambia, the barren Isles de Loss, the beautifully diversified features of Sierra Leone, the open and park-like territory around Accra, the low jungle-covered hills of Cape Coast Castle, and the rugged mountainous island of Fernando Po, however different in aspect, have all exhibited the same remarkable uniformity in giving birth to the disease." (1. Report, p. 26.)

This passage contains ample reasons for further research, by showing us that such familiar words as malaria or marshy miasma, if not hypothetical, are yet far from furnishing that absolute explanation of the origin of these and similar fevers which they have often been supposed to do. Still the general tenor of this report and information derived from other sources regarding the territory under consideration tend to show that this fever, though existing in various and diversified localities, is most prevalent and malignant where the character of the country and its vegetation would lead the believer in marshy effluvia to apprehend its assaults. In "the beautiful and diversified Sierra Leone," the deaths in two years were 650 per 1000 of the force employed; in "the barren Isles de Loss," 600 per 1000; but on "the low and swampy Gambia they were 1500 per 1000. "The sandy wastes of Senegal," it should be observed, too, are not without their marshes. "Remittent and intermittent fevers," says M. Thévenot, "dysentery, and nervous colic, these are the most formidable enemies of Europeans (in Senegal). The marshy soil of Senegal does not differ from that of Sologne, of Bresse, and of Aunis. The same causes produce the same effects. In both cases there is decomposition of organic substances, and poisoning of the air by marshy emanations, and the action of this air on men takes place in virtue of the same laws; but if the direct causes of the endemic maladies are in the two cases identically the same, it is not so with the subsidiary causes. There is at least such a difference in the intensity of their action, that patients receive from it an impression which often cannot be mistaken." (pp. 232-3).

We have no disposition to undervalue the opinion of Major Tulloch, formed, as is manifested by this and all his reports, from a survey at once minute and extensive of facts, and a careful deduction from them, and we think that he has shown that marshes, in the ordinary meaning of this term, are by no means essential to the production of the fevers in question, but he has not shown that they do not produce them, or that of all soils they are not the most calculated to produce them. When he quotes situations exempt from marsh as engendering these fevers, it will still be urged that vegetable decomposition is there possible and even probable, under a tropical sun and a humid atmosphere, and that such situations differ in their effects from marshes only in degree, and that this difference in degree is shown in their respective effects as set forth in his own pages. It may be urged that this argument is hypothetical, there being no proof of the decomposition of organized matter in such situations except from their influence on the human frame, and that to reason thus is reasoning in a circle. We admit this, yet does the hypothesis derive much probability from the effect in almost all climates of manifest marsh, and we think it one very difficult to disprove.

Before transferring our attention from this pestilential coast to climates more congenial to the European constitution, we shall briefly sketch the mortality from other diseases besides fever, observing merely that the class of intermittents there displays its usual characteristic, that of producing much inefficiency among the troops, and but little mortality. We find the total admissions for eighteen years to have been 948, and the deaths but eleven, or 1 in 86.

From diseases of the lungs there have been admitted but 56 per 1000 of mean strength, of which only 4·9 per 1000 were fatal, being in both cases a smaller proportion than in the United Kingdom, where the admissions are 148 and the deaths 7·7. The most marked exemption, the reporter observes, in this class is from inflammation of the lungs, by which 8 per 1000 have been attacked annually, though the usual proportion in most other colonies is from 30 to 40 per 1000. Consumption has also been comparatively rare, there having been admitted during the 18 years comprehended in the reports but 7 cases, of which 3 were fatal. In reference to this we are supplied with the *consolatory* reflection that the limited duration of human life on this coast would not perhaps in some instances afford time for so lingering a disease to become fully developed.

From diseases of the liver, described under the terms acute and chronic inflammation and jaundice, there were 150 admissions and 11 deaths, the proportion of deaths to admissions being 1 in 14, and the admissions being 82 and the deaths 6 per 1000 of mean strength, this class of diseases having been nearly four times more prevalent and fatal among the white troops in this station than in any other command of which the statistical details have yet been investigated.

From diseases of the stomach and bowels, the total admissions in the Sierra Leone command were 929, and the deaths 76, being in the proportion of 1 in 12; whilst the annual ratio per 1000 of mean strength was 504 admissions and 41·3 deaths. The Sierra Leone commissioners being of opinion that the large proportion of salt rations had mainly contributed to this sickness and mortality, an alteration was made in this respect, when, though the admissions remained the same, the mortality from these diseases was reduced to one tenth of its former amount—certainly a surprising reduction.

These diseases are much more fatal on the Cape Coast command than at Sierra Leone, more than one fourth of the troops having perished from them. The deaths from dysentery were at the former station 135, from diarrhœa 4, forming a total of 139 from these diseases of an entire mortality of 421, and being in the ratio of 220·6 of a mortality of 668·3 per 1000 of the mean strength.

We expected to observe some notice of diseases of the spleen in these reports from Western Africa, knowing from personal observation that they are very frequent sequels of the fevers both remittent and intermittent which prevail there. They are often, however, combined with diseases of the liver, which may account for their not appearing in the returns, but we have seen them uncomplicated in this country in patients who had suffered from fever on that coast.

We pass to the sickness and mortality among troops serving in the island of St. Helena.

This island is situated in the southern Atlantic, about 2000 miles from the American and 1200 from the African continent, between 15° and 16° of south latitude, and 5° and 6° of west longitude. It is about 10½ miles in length, 6¾ in breadth, and has a superficial extent of above 30,000 acres.

When viewed from a distance, it presents only a mass of abrupt rugged rocks, towering to the height of 2700 feet, apparently divested of vegetation, and in several parts broken into immense chasms. These rocks, however, are intersected by valleys of various extent, but all extremely contracted. At the extreme of one of these valleys, on the north-west or leeward side of the island, is James Town, the capital. Near this the country is wild and rugged, and presents but little vegetation; but in proportion to its distance from the sea it becomes less so, and the luxuriance of vegetation increases; most of the uplands are covered with ver dure, and at an elevation of nearly 1700 feet are two level tracts, called Longwood and Francis Plains, well-wooded, and affording good pasture. Various small streams intersect the island, but there is no marshy ground.

Though within 15 degrees of the line, the climate is not unhealthy, nor is it found debilitating to Europeans. The south-east trade-wind affords a steady breeze, which in these latitudes is rarely disturbed by storms and gales, and brings with it a canopy of clouds sufficient to afford shelter from the vertical rays of the sun. The temperature varies considerably according to the nature of the locality and different degrees of elevation. In James Valley, for instance, where the principal part of the troops are quartered, the thermometer during the summer months sometimes rises as high as 85°, and is generally about 80°; but at Plantation House, which enjoys an elevation of 1783 feet, the average at that season is much the same as in Great Britain. The average annual temperature in the former situation we find to be 75½°, and the annual range to be 15°. In the latter situation we find the mean annual temperature to be 69°, and the range to be 16¼. This is a very uniform temperature, but this equality is *said* to be counterbalanced by the circumstance that, owing to the nature of the localities, an individual may pass in a few minutes from the heat of the tropics to that of a temperate region. The difference between the high and low grounds with regard to moisture is still more remarkable. At James Town in the valley, in 1826, the number of rainy days during the year was 46, and 54 inches of rain fell; whilst at Plantation House, on the height, there were 178 rainy days, and 281·5 inches of rain fell.

Major Tulloch remarks that it could scarcely have been anticipated that, under the tropics, a situation could be found where the mortality among the white and black population did not, on the average of a long series of years, exceed that of their respective countries. He has shown, however, that such a situation has been found in St. Helena. Of a population amounting (exclusive of military) to an average of 3600, there died in 22 years 1627, being annually 74. According to this estimate the annual mortality has averaged one in 48½, even including the class termed strangers, many of whom landed in the last stage of disease; in the United Kingdom it averages about 1 in 47½ of the population; consequently St. Helena must be healthier than Britain.

According to the war-office returns, the deaths among the whole force in the island were 33 per 1000 annually, or, including 12 deaths at sea among invalids sent home for diseases contracted at St. Helena, 35 per 1000; but the medical returns, including troops of the line only, show the mortality in this force to have been but 25·4 per 1000 of the main strength annually. The admissions were fewer than among the most select force in the United Kingdom, in the proportion of 738 to 929; the diseases, however, were of a more dangerous character.

Above one third of the admissions and more than one half of the deaths arose from diseases of the stomach and bowels, which occurred in the proportion of 268, and were fatal in that of 13·9 per 1000 of mean strength annually. Of the admissions from this class nearly one half were from acute dysentery, and of the deaths three fourths arose from the same disease, which was fatal to one in 10⅘ of those attacked. The prevalence of bowel complaints generally and dysentery in particular is referred to the cause so often mentioned, salt rations.

Fevers are unfrequent and mild, the admissions of this class being but 78, and the deaths but 2·2 per 1000 of mean strength. Major Tulloch remarks, that there can be no better proof that this class of diseases may be comparatively rare, even within the tropics, than that the admissions have been fewer, in the proportion of 71 to 75, than among an equal force in the United Kingdom. Three fourths of the cases of this class are returned under the name of common continued fever.

As regards diseases of the lungs St. Helena is also remarkably healthy, the admissions from the whole of this class being but 61, and the deaths but 3·4 per 1000 annually; the admissions and deaths among the military being not half so high as in the United Kingdom or Mediterranean stations. The same feature is manifested in the fatal diseases of the population generally, in whom diseases of the lungs are fatal in the proportion of 3⅕ per 1000 annually. The exemption in this class is especially from inflammation of the lungs, which is the more remarkable, as a large proportion of the inhabitants are of the negro race, who in other climates manifest a great predisposition to this disease.

The admissions from liver disease have been 29 and the deaths 4 per 1000 annually of mean strength. To the total deaths which occur in the island, those from these diseases are in the proportion of 1 to 34½, whereas in England they are in the proportion of 1 to 78. This otherwise healthy climate, then, seems favorable to the production of diseases of the liver, resembling in this respect that of Western Africa. They occur, as the reporter remarks, even more frequently, and are of a graver character than in the West Indies, though the temperature is lower and more uniform, and though other diseases are more rare.

This seems the only real exception to the healthiness of this climate, so singular in a tropical latitude; for the prevalence of dysentery and other affections of the bowels does not appear attributable to climatorial influence.

The author considers the diseases of the Cape of Good Hope under two divisions, those of Cape Town and its vicinity, called the Cape districts, and those of the eastern frontier of the colony. We shall first give an abstract of his account of the former.

The Cape of Good Hope lies in lat. 34° 22′ south, long. 18° 24′ east. Cape Town is situated on a gravelly plain at the west side of Table Bay, having a gentle ascent to the foot of three barren precipitous mountains, which, stretching from the north-west to the north-east, form an amphitheatre, having its front to the bay. The soil is sandy, the under stratum rocky, and there is no marsh in the district. The maximum temperature as indicated by the thermometer is 86° and the minimum 56°, being a range of 30° throughout the year. This account, however, gives but an imperfect idea of the real temperature, which is very much augmented by the reflection of the sun's rays from the surface, composed principally of sandstone of the adjacent mountains already described. This reflection has raised a thermometer, which stood at 86° in the shade, to 136°. The average number of rainy days is 75, and the inches of rain are 41 throughout the year.

These are circumstances which, with the exception of the great heat, appear to promise health, and the promise is fulfilled. The admissions into hospital are numerous, being in the ratio of 991 per 1000 of mean strength; but two thirds of these are on account of diseases which seldom prove fatal, and the deaths are only in the proportion of 13·7 per 1000 of mean strength. The latter ratio, however, is exclusive of accidental deaths out of hospital. When these are added the mortality is found to be 15½ per 1000, or nearly the same as among dragoon guards and dragoons in the United Kingdom. The fevers appear to be mild, for though 88 per 1000 are admitted annually, of these 1 only in 45 is fatal, the proportion being little more than in the United Kingdom. By far the greater number of cases are returned under the head of common continued fever. Of diseases of the lungs there are 98 per 1000 admitted, of which 1 in 25 is fatal, or 3·9 per 1000 annually, a proportion much below the average of our colonies, those of the Mediterranean included. Diseases of the liver send 22 per 1000 to hospital, of which 1 in 20 is fatal. Of diseases of the stomach and bowels 126 per 1000 are admitted, and 3·1 per 1000 fatal. The ratio of admissions is lower than in British America, but with this difference, that dysentery is the prevailing form at the Cape, one half of the admissions arising from it, and whilst in the acute form it is fatal to one in 48½ of those attacked, a proportion pass into the chronic form, which is fatal to 1 in 4½. Diseases of the brain attack 10 per 1000 of the mean strength, and are fatal to 1 in 7·18 of those attacked, a degree of prevalence and severity nearly the same as in British America: a large proportion of the cases is said to arise from intemperance; but at the Cape, brain affection takes the form of delirium tremens in the proportion of only one tenth of the degree in which it prevails in North America. On this fact Major Tulloch makes the remark, that " If the relative prevalence of delirium tremens throughout all the colonies is investigated, it will be found rare wherever wine is procurable at a moderate rate, compared with stations where spirits form the principal intoxicating medium, a circumstance which should lead to the latter being placed under more rigid regulation than the former." (Cape Report, p. 10.)

We wish we could within our limits transfer to our pages many of the very curious details respecting the soil and climate of the eastern frontier district, and the connexion between circumstances ordinarily supposed to be the sources of insalubrity, and a high degree of healthfulness. The following is among the most striking of these details :

"On the Keiskamma and Great Fish Rivers, the thermometer about noon has been frequently known to range for several weeks from 105° to 110° in the shade, and from 135° to 140° in the sun, and even during several months it has seldom been under 95° at that hour. This portion of the colony, however, is throughout the whole year subject to very sudden transitions of temperature; the thermometer in summer has been known to fall from 110° to 64° in the course of a few hours ; and in winter, though it is often as low as the freezing point at night, it sometimes rises to 70° or 80° at midday. The degree of heat in summer is in a great degree regulated by the quantity of rain in the preceding season ; if the fall has been plentiful, the summer is comparatively cool; if scanty, the reverse. Notwithstanding the extremely high temperature of this climate, its salubrity is probably unequalled in any portion of the globe. As a proof, we may say, that in the three adjoining districts of Somerset, Albany, and Witenhage, the deaths in 1833 did not amount to more than 327 in a population of 30,000, being only 1 in 91, which is much lower than has ever been observed in the very healthiest districts of Great Britain." (Cape Report, 12 b.)

The report from the Eastern Frontier district shows an exemption from disease which we regard as unparalleled. The admissions into hospital are to those of troops in the United Kingdom as 866 to 929, and the deaths as 9·8 to 14. Diseases of the lungs are to those at home as 2·4 to 7·7, and only half as high in Malta, where the same dryness of atmosphere exists as in this singularly favoured district. The mortality from fever is 1·2 per 1000, whilst at Cape Town it is 1·9, and in the United Kingdom 1·4. No part of the world seems to enjoy such an exemption from fever, especially those of the intermittent and remittent types; and this is the case, though several of the stations are situated close to the bank of a river, which, being either dry or stagnant during summer, might be expected, under a high temperature, to give rise to exhalations such as are supposed to induce febrile diseases in the vicinity of *fumiares*, or beds of mountain-torrents in Spain, Portugal, and the Ionian Islands. Diseases of the brain are fewer than at home, though the troops are often exposed to a temperature which, during summer, generally ranges from 95° to 100° in the shade at midday.

It is remarkable that this very salubrious climate appears to agree better with the European than the native constitution, though healthful to all. The mortality among the Hottentot troops is 1·1 per 1000 higher than among the whites, being 10·9 per 1000 annually. This excess is chiefly owing to the greater tendency to pulmonary disease and affections of the bowels among the Hottentots than the white troops. Febrile diseases are exceedingly rare and mild among them ; so much so, that Major Tulloch remarks that it may be doubted whether any race of men in any quarter would be found to exhibit so great an exemption from them. The deaths from these diseases has been but 7 per 1000 of mean strength annually.

This singularly healthy colony manifests its character equally in the case of officers and men; the mortality in the former class being 14 per 1000 of the strength annually, including accidental deaths, in no way attributable to the climate. The ratio discharged as unfit for service from among the soldiers has amounted to 15 per 1000 of the force annually; being lower than in any of the other stations which have as yet been passed under review. Of those pensioned for various diseases, the proportion of persons sent home on account of pulmonic affections is much lower than from any other colony, showing that the influence of this class of diseases is especially small at the Cape.

We pass to the sickness and mortality among the troops serving in the Mauritius.

This island is of an irregular oval shape, 36 miles in length, and from 18 to 27 in breadth, with a superficial extent of nearly half a million of acres. It is situated in the Indian ocean, about 500 miles to the eastward of Madagascar, and from 70 to 80 north-east of the island of Bourbon, and lies in lat. 20° 9′ s., long. 57° 28′ e. The land rises rapidly from the coast to the interior, where it forms three chains of mountains from 1000 to 2000 feet in height. The country, except towards the summit of the mountains, is generally well wooded. The whole line of coast is well watered, from the foot of the mountains to the sea. The soil is in many parts exceedingly rich; but in the neighbourhood of Port Louis, and generally in the immediate vicinity of the sea, there is but a scanty covering of light friable soil over a rocky surface of coralline formation.

The maximum temperature at Port Louis is 88°, the minimum 70°; the former occurring in January and February, the latter in July and August: for Mauritius, lying to the southward of the equator, the seasons are there reversed. It will be understood that great variety of temperature is experienced according to the elevation attained; so that in the high regions of the interior, fires are often necessary. On an average of seven years, 39·30 inches of rain fell annually at Port Louis; but the humidity of localities is much influenced by the vicinity of mountains. It is observed that so far as regards temperature, rain, physical aspect, and diversity of climate, this island presents a striking resemblance to Jamaica.

According to an estimate, the data of which Major Tulloch states to be not very precise, the average annual mortality of the resident white population is 1 in 41 of both sexes, or rather less than in Malta. The more precise statistics of Mr. Thomas show that in the adjacent island of Bourbon, the mortality of the same class does not exceed 1 in 45, which is nearly the same as in the United Kingdom; a circumstance calculated to confirm the opinion of the general healthfulness of the Mauritius.

The sickness and mortality among the troops, however, are more considerable than this account of the white resident population would lead us to expect, there being admitted into hospital 1249, and dying (including deaths from violent and accidental causes) 30½ per 1000 of mean strength annually. Of these admissions and deaths, a very small proportion indeed, considering that the climate is a tropical one, arises from fever, the admissions being 154 per 1000 of mean strength, and the deaths being but 1·7, or 1 in 89 of those attacked; this is the average for 19 years, and that for the six preceding years exceeds it only by a very insignificant fraction. During the period of 19 years, the admissions from remittent fever were but 6, and of these 1 only died. Major Tulloch remarks on this, that Jamaica, at the same distance from the equator as the Mauritius, with a temperature identically the same, a similar mountainous elevation in the interior, little difference as to soil or moisture, and the same amount of marshy ground, has lost during the same period from this disease 5114, of a garrison larger by only about one-half than that of the former island. This is one of the numerous *nodi* with which Major Tulloch seems to delight in perplexing medical men.

It is in other classes of disease that we must seek for the excess in the mortality of the Mauritius, compared with the Cape. From diseases of the lungs 5·6 per 1000 die annually, or twice as many as at the Cape. This excess arises from the comparative prevalence of consumption, to which more than one half of the mortality from this class is due, and with which $7\frac{7}{10}$ of the mean strength is attacked annually; a higher proportion than in the United Kingdom, the Mediterranean, or even America—a proof that a mild and agreeable temperature and an insular situation furnish no exception from this disease. Inflammatory affections of the lungs by no means abound; another evidence, in addition to the proofs produced by Louis, that pneumonia and consumption do not abound contemporaneously, but rather present a contrast in prevalence in any locality.

Diseases of the liver attack 82, and are fatal to 4 per 1000 annually. Affections of the stomach and bowels attack 275, and destroy 10·6 per 1000 annually; and of these attacks five eighths and of the deaths seven eighths arise from dysentery. The first attack is said to be seldom fatal, examinations having shown the tissue of the bowels injured by former attacks in those who have died. The chief mortality from it is in soldiers advanced in life, the younger men suffering only to one fourth the extent of those above the age of forty. Many of the cases are connected with hepatic derangement, but in many no such connexion can be traced. Officers and white residents suffer much less from dysentery and other bowel complaints than soldiers.

Epidemic cholera appeared in this colony, as is well known to those acquainted with the history of this disease, in 1819. The admissions were 268, the deaths 32, or one in $8\frac{1}{3}$, a much lower proportion of mortality than occurred from the same cause in the United Kingdom, and other colonies. Major Tulloch says the disease exhibited nothing of a contagious character. He of course rejects the story of importation by the Topaze frigate.

Diseases of the brain gave rise to 41 admissions, and 2·7 deaths per 1000 of the mean strength annually. Five twelfths of these admissions and five-eighths of the deaths arise from *delirium tremens*, and the returns show that it has been progressively on the increase. The extent of intemperance at home and in our various colonies being traced by the test of this disease, it would appear to have attained its maximum in the Mauritius, *delirium tremens* being there the most prevalent, the Windward and Leeward Command in the West Indies alone competing with it in this bad preeminence. The cheapness of arrack furnishes an explanation of this unfortunate distinction. Whether there is anything special in the effect of this form of alcohol, we do not know.

After noticing the diseases of a detachment, furnished by one of the regiments stationed at Port Louis, to a group of islands called the Leychelles, but which call for no observation here, the author gives the details of the sickness and mortality in a corps of black pioneers in the Mauritius, which present some points of interest. This corps is composed of negroes either born in the Mauritius, or brought from Madagascar and Mozambique. These men are subject to no harsh treatment, their rations are abundant and suited to their tastes and constitutions, and they are described as a robust and athletic race. Yet do they die at the rate of 37·2 per 1000 annually, that is four times as rapidly as the inhabitants of the Cape, or other healthy countries at the same age, and at least thrice as rapidly as the white population of the Mauritius. The whole negro population of this island is decreasing so rapidly that in five years the deaths here exceeded the births by upwards of 6000 in a population of 60,000. It would seem that Mauritius and the West Indies are alike unsuited to the negro race : but it really does not appear on which of the many circumstances included in that comprehensive word, climate, the unsuitability depends. In countries so situated as Mauritius and the West Indies, it can scarcely depend on deficiency of temperature relatively to the negro constitution.

Diseases of the liver, stomach and bowels, and brain contribute considerably to this high rate of mortality, but diseases of the lungs are its greatest source, 12·9 per 1000 dying annually in this way, or more than twice as many as of the white troops. The following are the proportions in which negro troops die of diseases of the lungs at different stations : West Coast of Africa $6\frac{3}{10}$ per 1000, Honduras $8\frac{1}{10}$, Bahamas $9\frac{7}{10}$, Jamaica $10\frac{3}{10}$, Mauritius $12\frac{9}{10}$, Windward and Leeward Command $16\frac{5}{10}$, Gibraltar $33\frac{5}{10}$. In his native country the negro suffers from these diseases as British troops do at home, but after quitting it they affect him in a ratio varying in different colonies in a degree not explicable by the manifest circumstances of the climate, but always more considerably than in his native country. Consumption may be fairly estimated as causing two thirds of the numerous deaths from diseases of the lungs in negroes, yet natives of some tropical climates are so little prone to the disease, that among 74,850 native troops serving in the Madras Presidency, the deaths by every description of disease of the lungs did not, on the average of five years, exceed 1 per 1000 of the strength annually.

The deaths among the black troops from liver disease, is rather in a considerable ratio, that of 5·7 per 1000 of the strength. As the negro does not suffer to any extent from this disease, either on his native coast or in the West Indies, the reporter regards it as an evidence of some peculiar but unexplained tendency in the climate. May it not be regarded as part of a more general but equally unexplained tendency in the whole climate of the eastern hemisphere to generate disease of the liver?

We again take leave of Major Tulloch, and with an increase of our sentiments of respect. The additions which he has made to our knowledge of climates are alike honorable to his talent as a *redacteur*, and to that department of the public service whose very precise, voluminous, and long-accumulated treasures he has turned to such valuable account. We yield to no medical writer in *esprit de corps;* our "order" shall ever find us the unflinching advocates of their rights and privileges; but we do not regard ourselves as violating this sacred principle when we rejoice that the valuable treasures of the Army Medical Board Office were made over to a lay brother to be arranged in a form suitable to the compendious taste of the public. From experience of the manner in which questions of etiology have hitherto been treated by members of our body, we cannot help apprehending that the same amount of materials intrusted to more orthodox hands might have led to more of speculation and disputation, and less of practical usefulness than in those of the gallant Major. He has carefully eschewed hypothesis, and consequently avoided ground of controversy; but in precise practical facts relative to the climates treated of, his reports are unmatched by anything in our own, or, we believe, any other language. Some deductions towards the establishment of general facts or principles he could not avoid making; but those he produces seem rather calculated to lead us to these very desirable objects by showing the doubtful foundations of some of our present opinions, and thus clearing the ground for further investigation, than destined to form part of novel theories to be engendered by the writer. He manifests a greater disposition to show us that our present structures rest on frail ground than to become himself an architect.

The work of M. Thévenot contains a great mass of very precise statistical information regarding the French colonies in Western Africa, and had we considered them as likely to interest the British reader, our extracts from it should have been more abundant. As it is, we have availed ourselves of its contents chiefly to illustrate certain points in the corresponding parts of the reports. It is a book highly creditable to the writer, and it well merits the distinction conferred upon it, that of being published by order of the French Minister of Marine and Colonies.

Mr. Boyle's book is evidently the work of an experienced tropical practitioner. It is more strictly practical and less statistical than the other two works, but in its way one of great value. His descriptions of the diseases of Africa, particularly of the fevers, are excellent, and his practical suggestions bear all the stamp of observation and experience. We can conscientiously recommend his book as a safe tropical guide, and especially to the visitor of the Western Coast of Africa.

---

### Art. V.

*Medical and Physiological Commentaries.* By Martyn Paine, m.d., a.m. —*New York,* 1840. Two vols. 8vo, pp. 1531.

